# Supplementary material for: A fault-intrusion-tolerant system and deadline-aware algorithm for scheduling scientific workflow in the cloud
Source: PeerJ Comput Sci. 2021 Nov 2;7:e747. doi: 10.7717/peerj-cs.747 (PMC8576554; doi:10.7717/peerj-cs.747)
Supplement: Supplemental Information 6 [file peerj-cs-07-747-s006.zip › Myproject1.1/docs/org/cloudbus/cloudsim/CloudletSchedulerDynamicWorkload.html]

CloudletSchedulerDynamicWorkload


JavaScript is disabled on your browser.


- Overview
- Package
- Class
- Use
- Tree
- Deprecated
- Index
- Help

- Prev Class
- Next Class

- Frames
- No Frames

- All Classes

- Summary:
- Nested |
- Field |
- Constr |
- Method

- Detail:
- Field |
- Constr |
- Method


org.cloudbus.cloudsim

## Class CloudletSchedulerDynamicWorkload

- java.lang.Object
- - org.cloudbus.cloudsim.CloudletScheduler
  - - org.cloudbus.cloudsim.CloudletSchedulerTimeShared
    - - org.cloudbus.cloudsim.CloudletSchedulerDynamicWorkload

- ---

    

  ```
  public class CloudletSchedulerDynamicWorkload
  extends CloudletSchedulerTimeShared
  ```

  CloudletSchedulerDynamicWorkload implements a policy of scheduling performed by a virtual machine
  assuming that there is just one cloudlet which is working as an online service.

  Since:
  :   CloudSim Toolkit 2.0

- - ### Field Summary

    - ### Fields inherited from class org.cloudbus.cloudsim.CloudletSchedulerTimeShared

      `currentCPUs`
  - ### Constructor Summary

    Constructors

    Constructor and Description

    `CloudletSchedulerDynamicWorkload(double mips,
    int numberOfPes)`

    Instantiates a new vM scheduler time shared.
  - ### Method Summary

    Methods

    | Modifier and Type | Method and Description |
    | --- | --- |
    | `void` | `cloudletFinish(ResCloudlet rcl)` Processes a finished cloudlet. |
    | `double` | `cloudletSubmit(Cloudlet cl)` Receives an cloudlet to be executed in the VM managed by this scheduler. |
    | `double` | `cloudletSubmit(Cloudlet cl, double fileTransferTime)` Receives an cloudlet to be executed in the VM managed by this scheduler. |
    | `protected java.util.List<java.lang.Double>` | `getCacheCurrentRequestedMips()` Gets the cache current requested mips. |
    | `protected double` | `getCachePreviousTime()` Gets the cache previous time. |
    | `java.util.List<java.lang.Double>` | `getCurrentRequestedMips()` Gets the current mips. |
    | `double` | `getEstimatedFinishTime(ResCloudlet rcl, double time)` Get estimated cloudlet completion time. |
    | `double` | `getMips()` Gets the mips. |
    | `int` | `getNumberOfPes()` Gets the pes number. |
    | `double` | `getTotalCurrentAllocatedMipsForCloudlet(ResCloudlet rcl, double time)` Gets the current mips. |
    | `double` | `getTotalCurrentAvailableMipsForCloudlet(ResCloudlet rcl, java.util.List<java.lang.Double> mipsShare)` Gets the total current mips for the clouddlet. |
    | `int` | `getTotalCurrentMips()` Gets the total current mips. |
    | `double` | `getTotalCurrentRequestedMipsForCloudlet(ResCloudlet rcl, double time)` Gets the current mips. |
    | `double` | `getTotalMips()` Gets the total mips. |
    | `double` | `getTotalUtilizationOfCpu(double time)` Get utilization created by all cloudlets. |
    | `java.util.Map<java.lang.String,java.lang.Double>` | `getUnderAllocatedMips()` Gets the under allocated mips. |
    | `protected void` | `setCacheCurrentRequestedMips(java.util.List<java.lang.Double> cacheCurrentRequestedMips)` Sets the cache current requested mips. |
    | `protected void` | `setCachePreviousTime(double cachePreviousTime)` Sets the cache previous time. |
    | `void` | `setMips(double mips)` Sets the mips. |
    | `void` | `setNumberOfPes(int pesNumber)` Sets the pes number. |
    | `void` | `setTotalMips(double mips)` Sets the total mips. |
    | `void` | `setUnderAllocatedMips(java.util.Map<java.lang.String,java.lang.Double> underAllocatedMips)` Sets the under allocated mips. |
    | `void` | `updateUnderAllocatedMipsForCloudlet(ResCloudlet rcl, double mips)` Update under allocated mips for cloudlet. |
    | `double` | `updateVmProcessing(double currentTime, java.util.List<java.lang.Double> mipsShare)` Updates the processing of cloudlets running under management of this scheduler. |

    - ### Methods inherited from class org.cloudbus.cloudsim.CloudletSchedulerTimeShared

      `cloudletCancel, cloudletPause, cloudletResume, getCapacity, getCloudletExecList, getCloudletFinishedList, getCloudletPausedList, getCloudletStatus, getCurrentRequestedUtilizationOfBw, getCurrentRequestedUtilizationOfRam, getNextFinishedCloudlet, isFinishedCloudlets, migrateCloudlet, runningCloudlets, setCloudletExecList, setCloudletFinishedList, setCloudletPausedList`
    - ### Methods inherited from class org.cloudbus.cloudsim.CloudletScheduler

      `getCurrentMipsShare, getPreviousTime, setCurrentMipsShare, setPreviousTime`
    - ### Methods inherited from class java.lang.Object

      `clone, equals, finalize, getClass, hashCode, notify, notifyAll, toString, wait, wait, wait`

- - ### Constructor Detail


    - #### CloudletSchedulerDynamicWorkload

      ```
      public CloudletSchedulerDynamicWorkload(double mips,
                                      int numberOfPes)
      ```

      Instantiates a new vM scheduler time shared.

      Parameters:
      :   `mips` - the mips
      :   `numberOfPes` - the pes number
  - ### Method Detail


    - #### updateVmProcessing

      ```
      public double updateVmProcessing(double currentTime,
                              java.util.List<java.lang.Double> mipsShare)
      ```

      Updates the processing of cloudlets running under management of this scheduler.

      **Overrides:**
      :   `updateVmProcessing` in class `CloudletSchedulerTimeShared`

      Parameters:
      :   `currentTime` - current simulation time
      :   `mipsShare` - array with MIPS share of each Pe available to the scheduler

      Returns:
      :   time predicted completion time of the earliest finishing cloudlet, or 0 if there is
          no next events


    - #### cloudletSubmit

      ```
      public double cloudletSubmit(Cloudlet cl)
      ```

      Receives an cloudlet to be executed in the VM managed by this scheduler.

      **Overrides:**
      :   `cloudletSubmit` in class `CloudletSchedulerTimeShared`

      Parameters:
      :   `cl` - the cl

      Returns:
      :   predicted completion time


    - #### cloudletSubmit

      ```
      public double cloudletSubmit(Cloudlet cl,
                          double fileTransferTime)
      ```

      Receives an cloudlet to be executed in the VM managed by this scheduler.

      **Overrides:**
      :   `cloudletSubmit` in class `CloudletSchedulerTimeShared`

      Parameters:
      :   `cl` - the cl
      :   `fileTransferTime` - the file transfer time

      Returns:
      :   predicted completion time


    - #### cloudletFinish

      ```
      public void cloudletFinish(ResCloudlet rcl)
      ```

      Processes a finished cloudlet.

      **Overrides:**
      :   `cloudletFinish` in class `CloudletSchedulerTimeShared`

      Parameters:
      :   `rcl` - finished cloudlet


    - #### getTotalUtilizationOfCpu

      ```
      public double getTotalUtilizationOfCpu(double time)
      ```

      Get utilization created by all cloudlets.

      **Overrides:**
      :   `getTotalUtilizationOfCpu` in class `CloudletSchedulerTimeShared`

      Parameters:
      :   `time` - the time

      Returns:
      :   total utilization


    - #### getCurrentRequestedMips

      ```
      public java.util.List<java.lang.Double> getCurrentRequestedMips()
      ```

      Gets the current mips.

      **Overrides:**
      :   `getCurrentRequestedMips` in class `CloudletSchedulerTimeShared`

      Returns:
      :   the current mips


    - #### getTotalCurrentRequestedMipsForCloudlet

      ```
      public double getTotalCurrentRequestedMipsForCloudlet(ResCloudlet rcl,
                                                   double time)
      ```

      Gets the current mips.

      **Overrides:**
      :   `getTotalCurrentRequestedMipsForCloudlet` in class `CloudletSchedulerTimeShared`

      Parameters:
      :   `rcl` - the rcl
      :   `time` - the time

      Returns:
      :   the current mips


    - #### getTotalCurrentAvailableMipsForCloudlet

      ```
      public double getTotalCurrentAvailableMipsForCloudlet(ResCloudlet rcl,
                                                   java.util.List<java.lang.Double> mipsShare)
      ```

      Gets the total current mips for the clouddlet.

      **Overrides:**
      :   `getTotalCurrentAvailableMipsForCloudlet` in class `CloudletSchedulerTimeShared`

      Parameters:
      :   `rcl` - the rcl
      :   `mipsShare` - the mips share

      Returns:
      :   the total current mips


    - #### getTotalCurrentAllocatedMipsForCloudlet

      ```
      public double getTotalCurrentAllocatedMipsForCloudlet(ResCloudlet rcl,
                                                   double time)
      ```

      Gets the current mips.

      **Overrides:**
      :   `getTotalCurrentAllocatedMipsForCloudlet` in class `CloudletSchedulerTimeShared`

      Parameters:
      :   `rcl` - the rcl
      :   `time` - the time

      Returns:
      :   the current mips


    - #### updateUnderAllocatedMipsForCloudlet

      ```
      public void updateUnderAllocatedMipsForCloudlet(ResCloudlet rcl,
                                             double mips)
      ```

      Update under allocated mips for cloudlet.

      Parameters:
      :   `rcl` - the rgl
      :   `mips` - the mips


    - #### getEstimatedFinishTime

      ```
      public double getEstimatedFinishTime(ResCloudlet rcl,
                                  double time)
      ```

      Get estimated cloudlet completion time.

      Parameters:
      :   `rcl` - the rcl
      :   `time` - the time

      Returns:
      :   the estimated finish time


    - #### getTotalCurrentMips

      ```
      public int getTotalCurrentMips()
      ```

      Gets the total current mips.

      Returns:
      :   the total current mips


    - #### setTotalMips

      ```
      public void setTotalMips(double mips)
      ```

      Sets the total mips.

      Parameters:
      :   `mips` - the new total mips


    - #### getTotalMips

      ```
      public double getTotalMips()
      ```

      Gets the total mips.

      Returns:
      :   the total mips


    - #### setNumberOfPes

      ```
      public void setNumberOfPes(int pesNumber)
      ```

      Sets the pes number.

      Parameters:
      :   `pesNumber` - the new pes number


    - #### getNumberOfPes

      ```
      public int getNumberOfPes()
      ```

      Gets the pes number.

      Returns:
      :   the pes number


    - #### setMips

      ```
      public void setMips(double mips)
      ```

      Sets the mips.

      Parameters:
      :   `mips` - the new mips


    - #### getMips

      ```
      public double getMips()
      ```

      Gets the mips.

      Returns:
      :   the mips


    - #### setUnderAllocatedMips

      ```
      public void setUnderAllocatedMips(java.util.Map<java.lang.String,java.lang.Double> underAllocatedMips)
      ```

      Sets the under allocated mips.

      Parameters:
      :   `underAllocatedMips` - the under allocated mips


    - #### getUnderAllocatedMips

      ```
      public java.util.Map<java.lang.String,java.lang.Double> getUnderAllocatedMips()
      ```

      Gets the under allocated mips.

      Returns:
      :   the under allocated mips


    - #### getCachePreviousTime

      ```
      protected double getCachePreviousTime()
      ```

      Gets the cache previous time.

      Returns:
      :   the cache previous time


    - #### setCachePreviousTime

      ```
      protected void setCachePreviousTime(double cachePreviousTime)
      ```

      Sets the cache previous time.

      Parameters:
      :   `cachePreviousTime` - the new cache previous time


    - #### getCacheCurrentRequestedMips

      ```
      protected java.util.List<java.lang.Double> getCacheCurrentRequestedMips()
      ```

      Gets the cache current requested mips.

      Returns:
      :   the cache current requested mips


    - #### setCacheCurrentRequestedMips

      ```
      protected void setCacheCurrentRequestedMips(java.util.List<java.lang.Double> cacheCurrentRequestedMips)
      ```

      Sets the cache current requested mips.

      Parameters:
      :   `cacheCurrentRequestedMips` - the new cache current requested mips


- Overview
- Package
- Class
- Use
- Tree
- Deprecated
- Index
- Help

- Prev Class
- Next Class

- Frames
- No Frames

- All Classes

- Summary:
- Nested |
- Field |
- Constr |
- Method

- Detail:
- Field |
- Constr |
- Method
